# Prolonged SARS-CoV-2 RNA Shedding from Therapy Cat after Cluster Outbreak in Retirement Home

**DOI:** 10.3201/eid2707.204670

**Published:** 2021-07

**Authors:** Claudia Schulz, Claudia Wylezich, Kerstin Wernike, Magdalena Gründl, Alexandra Dangel, Christine Baechlein, Donata Hoffmann, Susanne Röhrs, Sabrina Hepner, Nikolaus Ackermann, Andreas Sing, Isabelle Pink, Beate Länger, Holger A. Volk, Paul Becher, Gerd Sutter, Antonie Neubauer-Juric, Maren von Köckritz-Blickwede, Martin Beer, Asisa Volz

**Affiliations:** University of Veterinary Medicine Hanover, Hanover, Germany (C. Schulz, C. Baechlein, S. Röhrs, B. Länger, H.A. Volk, P. Becher, M. von Köckritz-Blickwede, A. Volz);; Friedrich-Loeffler-Institut, Greifswald-Insel Riems, Germany (C. Wylezich, K. Wernike, D. Hoffmann, M. Beer);; Local Health Authority Cham, Cham, Germany (M. Gründl);; Bavarian Health and Food Safety Authority, Oberschleißheim, Germany (A. Dangel, S. Heppner, N. Ackermann, A. Sing, A. Neubauer-Juric);; Hanover Medical School, Hanover (I. Pink); Ludwig Maximilian University Munich, Munich, Germany (G. Sutter)

**Keywords:** severe acute respiratory syndrome coronavirus 2, SARS-CoV-2, coronaviruses, viruses, coronavirus disease, COVID-19, respiratory infections, pandemic, cluster outbreak, RNA, shedding, therapy cat, animal reservoir, retirement home, zoonoses, Germany

## Abstract

We report a therapy cat in a nursing home in Germany infected with severe acute respiratory syndrome coronavirus 2 during a cluster outbreak in the home residents. Although we confirmed prolonged presence of virus RNA in the asymptomatic cat, genome sequencing showed no further role of the cat in human infections on site.

Cats are susceptible to severe acute respiratory syndrome coronavirus 2 (SARS-CoV-2) infection and can transmit the virus to other cats (*1**–**3*). However, the pathophysiology and epidemiologic impact of SARS-CoV-2 infection of pets remain poorly understood (*4*). We report 3 therapy cats living in a retirement home in Germany for which evidence indicated naturally occurring human-to-cat transmission during SARS-CoV-2 outbreaks.

A total of 21 confirmed human SARS-CoV-2 infections occurred in the outbreaks, including 3 deaths. Six infected care and administrative personnel showed mild or no symptoms; 15 infected residents showed typical signs of coronavirus disease, including fever and severe respiratory disease (cough, pneumonia, and dyspnea). The first outbreak occurred on the home’s ground floor at the end of March 2020 (Appendix Figure 1, panel A); it is assumed that the virus was introduced through care personnel. One SARS-CoV-2‒positive resident (90 years of age, given a diagnosis on April 4, 2020), already bedridden, died on April 12. He had been in close contact with cat K8, which snuggled in his face.

A strict hygienic plan was implemented to contain the initial outbreak, including using separate personnel for each floor. No visitors were allowed. All residents were kept in their rooms without social contact between them. Despite isolation, the cats still had access to all areas and to the outside. 

At the end of April, residents of the first floor showed typical COVID-19 symptoms. We tested oropharyngeal swab specimens from the cats on April 29 (surveillance day 1) (Appendix Figure 1, panel A). Although 2 cats (K4 and K9) showed negative results, 1 (K8) showed positive results for SARS-CoV-2 RNA by quantitative reverse transcription PCR specific for partial envelope protein gene (Table; Appendix Figure 1, panel C). 

Because of epidemiologic connections, we speculated whether K8 could have been involved in spreading SARS-CoV-2 to the first floor. We isolated the cats in a Biosafety Level 3 facility for surveillance (Appendix Figure 1, panel A) and tested them again on May 4. K8 was positive for SARS-CoV-2 RNA and had lower quantification cycle values (Table; Appendix Figure 1, panels B, C). Cats were housed in single cages during the first 4 days of quarantine (surveillance days 6‒10), then moved into 1 combined cage system (surveillance day 11). After 15 days (surveillance day 21), cats were transferred to floor housing under Biosafety Level 3 conditions and permitted free movement and contact. Testing at regular intervals of conjunctival, fecal, and oropharyngeal swab specimens showed that K4 and K9 remained negative, whereas K8 was positive for SARS-CoV-2 RNA until day 21 of surveillance. K8 also had positive quantification cycle values (range 26.3‒38.5; values <40 were considered positive) (Appendix Figure 1, panel B) and ≈5.7 ×10^4^ to 5.0 ×10^3^ RNA copies/mL (Appendix Figure 1, panel C).

**Table:** Overview of cat swab specimen and blood sampling regimen for prolonged SARS-CoV-2 RNA shedding from therapy cat after cluster outbreak in retirement home, Germany*

Day of surveillance	Sample type	Cq values by RdRp/RdRp gene screen	Cq values by E/E/S gene screen
1	OPS	ND/ND	ND/35.56/36.15†
6	OPS	ND/ND	ND/26.34/27.06†
7	OPS	29.66/31.40	30.52/ND
9	OPS, CS, FS, BS	37.48/no Cq	36.23/ND
11	OPS, CS, FS	30.95/34.80	34.66/ND
13	OPS, CS, FS, BS	32.63/35.50	35.98/ND
15	OPS, CS, FS	31.17/35.10	36.96/ND
17	OPS, CS, FS, BS	33.12/36.50	35.92/ND
19	OPS, CS, FS	No Cq/no Cq	No Cq/ND
21	OPS, CS, FS, BS	No Cq/38.80	No Cq/ND
**28**	OPS, CS, FS, **BS**	No Cq/no Cq	ND/ND
31	OPS, CS, FS	No Cq/no Cq	No Cq/ND
35	OPS, CS, FS, BS	No Cq/no Cq	ND/ND
38	OPS, CS, FS	No Cq/no Cq	ND/ND
42	OPS, CS, FS, BS	No Cq/no Cq	ND/ND
45	OPS, CS, FS	No Cq/no Cq	ND/ND
49	OPS, CS, FS, BS	No Cq/no Cq	ND/ND
69	BS	No Cq/no Cq	ND/ND
73	OPS, CS, FS, BS	No Cq/no Cq	ND/ND

*Results are given for virus-positive cat K8. At day 28, serum samples for analysis of blood were collected (in bold). PCRs were performed between day 1 and day 6 by using the RealStar SARS-CoV-2 RT-PCR Kit 1.0 (Altona Diagnostics, https://www.altona-diagnostics.com) for initial diagnosis (E gene screen and SARS-CoV-2 specific spike gene), and from day 7 by using an RdRP gene SARS-2-IP4 quantitative real-time PCR (World Health Organization–recommended assay) and an E gene-specific PCR (see details in Appendix). The 2 results in the RdRp and E gene columns indicate that these assays were performed independently in 2 different laboratories (University of Veterinary Medicine Hannover and Friedrich-Loeffler Institut). BS, blood sample; CS, conjunctival swab; Cq, quantification cycle; E, envelope; FS, fecal swab; ND, not done; OPS, oropharyngeal swab; RdRP, RNA-dependent RNA polymerase; S, spike protein; SARS-CoV-2, severe acute respiratory syndrome coronavirus 2. †Indicates a third test result for the S gene.

Subsequently, we detected no viral RNA in swab samples through day 73. 

These PCR results demonstrated an extended period of SARS-CoV-2 infection of the positive cat. When serum samples were analyzed for SARS-CoV-2‒neutralizing antibodies (*5*), K8 showed a positive titer (range 1:20‒1:52) (Appendix Figure 1, panel D). Multispecies ELISA results showed serum antibodies against the receptor-binding domain (*5*). Titers peaked by day 35 and decreased but remained positive until the end of surveillance (Appendix Figure 1, panel E). K4 and K9 remained SARS-CoV-2 seronegative (Appendix Figure 1, panels D, E).

To examine the effects of potential co-infections, we analyzed common feline viral infections. All cats were negative for feline leukemia virus. However, K8 was positive for feline immunodeficiency virus (FIV)–specific antibodies, and K4 and K9 were positive for feline coronavirus‒specific antibodies. The marginal serologic reactivity of K8 indicated that this cat was not previously infected with feline coronavirus (Appendix Figure 2).

SARS-CoV-2 genome sequences obtained from K8 and related human cases in the retirement home (1 from the first outbreak and 3 from the second outbreak) differed from each other by 3 ambiguous sites, indicating low-frequency variants within K8, leading to viral quasispecies. Sequences from the second outbreak included a constant C→T change (Appendix Figure, panel F). These data support direct human-to-cat-transmission during the first outbreak but not zoonotic SARS-CoV-2 transmission from K8 because of the constant viral sequence difference within the second outbreak series.

Our data showed human-to-cat SARS-CoV-2 transmission in a community-acquired cluster outbreak that had multiple infection events. We demonstrated prolonged shedding of SARS-CoV-2 RNA up to day 21 after the first detection, in contrast to a recent study in a naturally infected cat (RNA-positive for 11 days) (*6*). We hypothesize a longer period of RNA shedding (>21 days) because we do not know the day of infection before the start of cat surveillance. Prolonged SARS-CoV-2 RNA shedding could be related to immune status of individual animals or co-infections or immunosuppression as reported for humans (*7**–**9*).

Our sequencing data do not suggest zoonotic spillback from the SARS-CoV-2‒infected cat to humans, as reported elsewhere (*3**,**10*). However, reinfections, prolonged virus replication, and transmission events in cats cannot be excluded, in particular if one considers emergence of SARS-CoV-2 variants that have potentially increased host range or ability to escape preexisting immunity. Thus, cats should be considered in surveillance and control measures.

Appendix Additional information on prolonged SARS-CoV-2 RNA shedding from therapy cat after cluster outbreak in retirement home.
