# Supplementary material for: Prolonged SARS-CoV-2 RNA Shedding from Therapy Cat after Cluster Outbreak in Retirement Home
Source: Emerg Infect Dis. 2021 Jul;27(7):1974–6. doi: 10.3201/eid2707.204670 (PMC8237873; doi:10.3201/eid2707.204670)
Supplement: Appendix — Additional information on prolonged SARS-CoV-2 RNA shedding from therapy cat after cluster outbreak in retirement home. [file 20-4670-Techapp-s1.pdf]

Article DOI: https://doi.org/10.3201/eid2707.204670

Prolonged SARS-CoV-2 RNA Shedding from Therapy Cat after Cluster Outbreak in Retirement Home

Appendix

Detailed Description of COVID-19 Cluster Outbreak in Retirement Home

At the end of March 2020 (March 26–29), the first severe acute respiratory syndrome coronavirus 2 (SARS-CoV-2) infections were diagnosed in a retirement home in southern Germany. There were 21 laboratory-confirmed SARS-CoV-2–positive persons over a period of 10 weeks (Appendix Figure 1, panel A). On April 1, a 90-year-old woman who had been given a diagnosis of coronavirus disease (COVID-19) on March 30, 2020, died after showing severe clinical symptoms. A second patient (78-year-old man) who had been confirmed as being SARS-CoV-2 positive on April 4 died on April 8. There was still not a single case of infection on the first floor. On April 12, another male SARS-CoV-2 patient (90 years of age) who had been given a diagnosis on April 4 died because of COVID-19 (Appendix Figure 1, panel A). Because of underlying health conditions, this patient had already been bed-ridden before his disease, and had typical COVID-19 symptoms. This patient (owner of cat K8) had been in close contact with cat K8 for the entire period of the COVID-19 outbreak. There was no evidence for COVID-19 disease events on the first floor.

Protective clothing and rigorous isolation were used to further contain the initial outbreak. This included cohort isolation of the positive cases in one wing of the floor, including installing a gate and restricting access to only a few allocated care personnel who took care of the virus-positive patients. No visitors were allowed. In doing so, the first floor appeared to be spared from any SARS-CoV-2 infection. Despite the strict isolation, the cats still had access to all areas and to the outside. Because of the specific protective measures, all residents and staff were tested for SARS-CoV-2 at specific time intervals, which were set by the local health authority. It seemed that the initial SARS-CoV-2 outbreak could be stopped. However, second outbreak started at the end of April, when the first residents of the first floor showed typical

COVID-19 symptoms, including fever (Appendix Figure 1, panel A). On April 22, a 92-year-old woman was hospitalized. She was given a diagnosis of infection with SARS-CoV-2 on April 23. Up until April 27, all residents and staff had been tested for SARS-CoV-2.

In the context of the epidemiologic investigation of this second outbreak, the 3 therapy cats were also tested for SARS-CoV-2 by using oropharyngeal swab specimens. Cat K8, the close companion of the deceased bed-ridden COVID-19 patient from April 12, was confirmed to be SARS-CoV-2 positive by quantitative real-time quantitative reverse transcription PCR (qRT-PCR) on April 29 (Table; Appendix Figure 1, panel B). At this stage, 5 additional residents from the first floor were given diagnoses of COVID-19.

According to the SARS-CoV-2 genome information we could obtain for cat K8 and from 1 and 3 residents from the first and second outbreaks, respectively, we assume a separate entering of SARS-CoV-2 for the second outbreak in the care facility (on the basis of a nucleotide difference at sequence position 21157; Appendix Figure 1, panel F). Cat-specific mutations could not be identified for comparing nucleotide sequence of cat K8 with a cat sequence from the National Center for Biotechnology Information (Bethesda, MD, USA) NCBI (accession no. MT747438) that does not show any similar sequence variants.

Material and Methods

Ethics for Use of Animals

All animal studies were conducted in compliance with the German Regulations for Animal Experimentation (#20A522, Animal Welfare Act, approved by the Niedersächsisches Landesamt für Verbraucherschutz und Lebensmittelsicherheit (Lower Saxony, Germany). All animal and laboratory work was performed in a Biosafety level 3 (BSL-3) laboratory or a BSL-2 laboratory and facilities at the Research Center for Emerging Infections and Zoonoses, University of Veterinary Medicine, Hannover.

The 3 therapy cats monitored in this study (K4, female, ≈14 years of age; K8, female, ≈6 years of age; K9, male, ≈10 years of age) were obtained from a home for the elderly. In agreement with the management team of the senior resident’s home and the responsible veterinary authority, the cats were transferred to the BSL-3 animal facility at the University of Veterinary Medicine Hannover for detailed follow-up. Cats had free access to water and food.

Cats were monitored daily for well-being, health constitution, and clinical signs, such as body temperature, anorexia, diarrhea/loose stool, vomiting, lethargy/depression, and respiratory symptoms. Weights of all cats were checked daily. After repeated PCR confirmation that no SARS-CoV-2 RNA was excreted from any of the 3 cats, they were housed under BSL-2 conditions for another 14 days. After all cats were confirmed negative for SAR-CoV-2 by using PCR analysis, and they were discharged from the animal facility and placed again in private homes.

Ethics for Use of Human Samples

All participants and respective authorized person provided written informed consent. The study design was reviewed and approved by the institutional Ethics Review Board at the Hannover Medical School (#9350_BO_K2020).

Cat Sampling

Cats were sampled by using oropharyngeal swab specimens until day 7 of surveillance. From day 7 on, surveillance cats were sampled every other day (Table, Appendix Figure 1, panels B–E) by using oropharyngeal, rectal, and conjunctival swab specimens until day 21. After day 21 of surveillance, the cats were sampled by using oropharyngeal, rectal, and conjunctival swab specimens twice a week. After day 49 of surveillance, sampling was curtailed to a final sampling by oropharyngeal, rectal and conjunctival swab specimens at day 73 of surveillance. Swab specimens were placed immediately into a sterile transport tube containing 2 mL of Opti-MEM (Opti-MEM I Reduced Serum Medium, GlutaMAX Supplement, #51985026; ThermoFisher, https://www.thermofisher.com) containing 1% penicillin/streptomycin and stored at −80°C within 4 hours after collection until further analysis. Blood samples were taken at most twice a week from the cephalica or saphena vein. Serum samples were collected on days 9, 13, 17, 21, 28, 35, 42, 49, 69, and 73 of surveillance. EDTA was added to blood collected at day 28 of surveillance for blood cell analysis.

Human Sampling

Samples from humans were collected and tested by qRT-PCR as part of routine clinical care. Humans were sampled by using naso-oropharyngeal swab specimens from the nostril parallel to the palate to the nasopharynx with a gentle rub and roll. Swab specimens were immediately placed into sterile transport tubes containing 2–3 mL of virus transport medium.

Detection of Feline Leukemia Virus IgG

IgG for this virus was detected by using a qualitative ELISA (IDEXX Laboratories, https://www.idexx.com).

Detection of Feline Immunodeficiency Virus IgG

IgG for this virus was detected by using a qualitative ELISA (IDEXX Laboratories, https://www.idexx.com).

ELISA for Detection of Feline Coronavirus

Antigen-specific IgG responses for this virus were analyzed by using a qualitative ELISA (IDEXX Laboratories). For evaluation of test results, we calculated a quotient to determine specific cutoff values, and positive and negative results.

SARS-CoV-2 Neutralization Test and Indirect Multispecies ELISA

Serum samples were tested for SARS-CoV-2 neutralizing antibodies by using a virus neutralization assay (*1*). In brief, 50 μL of medium containing $10^{3.3}$ mean (50%) tissue culture infectious doses//mL of SARS-CoV-2 were mixed with 50 μL of diluted cat serum. Each sample was tested in triplicate. After 1 h of incubation at 37°C, the virus-serum mixture was transferred to confluent Vero E6 cells in a 96-well plate. Viral replication was assessed after incubation for 5 days at 37°C in an atmosphere of 5% CO2 by detection of a cytopathic effect.

Receptor-binding domain antibodies were detected by using a multispecies ELISA described by Wernike et al. (*2*). In brief, receptor-binding domain antigen–coated plates were incubated at 4°C overnight and blocked for 1 h at 37°C with 5% skim milk in phosphate-buffered saline. Serum samples were added and incubated for 1 h at room temperature, and a multispecies conjugate (SBVMILK; IDvet, https://www.id-vet.com) France) was added for 1 h at room temperature. Plates were washed 3 times after each step. After adding 3,3′,5,5′-tetramethylbenzidine substrate (IDEXX Laboratories), the ELISA result was read at a wavelength of 450 nm. Adsorbance was calculated by subtracting the optical density of uncoated wells from those of protein-coated wells for the respective sample (*2*).

Quantitative Real-Time Reverse Transcription PCR

Initial SARS-CoV-2 diagnosis for cats (days 1–6 of surveillance) and humans was performed by using the RealStar SARS-CoV-2 RT-PCR Kit 1.0 (#821005; Altona Diagnostics, https://www.altona-diagnostics.com) using the envelope (E) gene screening (lineage B

betacoronavirus) and SARS-CoV-2 specific (spike gene) assays. During cat monitoring at the containment animal facility at the University of Veterinary Medicine Hanover, nasal, oropharyngeal, and rectal swab specimens were stored in Opti-MEM (Opti-MEM I Reduced Serum Medium, GlutaMAX Supplement; #51985026; ThermoFisher) containing 1% penicillin/streptomycin at −80°C within 4 hours after collection until further analysis. RNA was extracted from a 140-µL sample by using the Qiamp Viral RNA Mini Kit (QIAGEN, https://www.qiagen.com) and eluted in 60 µL of AVE buffer according to the manufacturer's protocol. Furthermore, an internal control system and negative controls were used according to Jendrny et al. (*3*), Hoffmann (*4*), and Hoffmann et al. (*5*).

For SARS-CoV-2 RNA amplification, the AgPath-ID One-Step RT-PCR Kit (ThermoFisher) was used. SARS-CoV-2 RNA isolated from cell culture supernatants (kindly provided by Sven Reiche, Friedrich-Loeffler-Institut, Insel Riems, Germany) served as a positive control. SARS-CoV-2 RNA was amplified with the AriaMx Real-Time PCR (qPCR) Instrument (Agilent, https://www.agilent.com) by using a temperature profile of 10 min at 45°C and 10 min at 95°C, and 42 repetition cycles of 15 s at 95°C, 20 s at 57°C, and 30 s at 72°C described by Hoffmann (*4*). Therefore, the qRT-PCR specific for the RNA-dependent RNA polymerase gene of SARS-CoV-2 (SARS-2-IP4 assay, Institute Pasteur, 2020, assay recommended by the World Health Organization (*6*) was conducted as described by Jendrny et al. (*3*) and Hoffmann (*4*). Samples were analyzed collaterally in 2 independent laboratories (Friedrich-Loeffler-Institut, Insel Riems, Germany and University of Veterinary Medicine, Hanover, Germany). In addition, positive samples were analyzed and quantified for SARS-CoV-2 RNA copies/mL in another independent laboratory at the Institute for Virology, University of Veterinary Medicine Hanover, for confirmation and quantification of SARS-CoV-2 RNA. A qRT-PCR assay specific for the envelope (E) gene (E_Sarbeco assay) according to Corman et al. (*7*) was used.

For quantification, an RNA copy standard was used. In brief, SARS-CoV-2 RNA isolated from cell culture supernatants (kindly provided by Sven Reiche, Friedrich-Loeffler-Institut, Insel Riems, Germany) served to synthesize cDNA with random primers and Moloney Murine Leukemia Virus Reverse Transcription (ThermoFisher). Complementary DNA served to generate a 113 bp–spanning amplicon of the E gene by using Taq polymerase (ALLin HS Red Taq Mastermix; HighQu, https://www.highqu.com). The PCR product was TOPO cloned into the vector pCR2.1 under the transcriptional control of the T7 promoter (pCR2.1.SARS-COV-2-

E). Plasmid DNA integrity was confirmed by Sanger sequencing (LGS Genomics, https://shop.lgcgenomics.com). RNA transcripts were produced by using T7RNA polymerase and purified (MEGAscript T7 Transcription Kit and MEGAclear Clean-Up Kit, ThermoFisher). The synthetic RNAs were used to generate a standard dilution series for the qRT-PCR (10^6–10^1 copies). E gene-specific qRT-PCR E_Sarbeco assay (*7*) detected SARS-CoV-2 RNA with high sensitivity. Up to 10 RNA copies per reaction were successfully detected. RNA samples and RNA dilutions were run in parallel, and genome equivalents were calculated on the basis of Cq values obtained by amplification of the RNA copy standard.

Next-Generation Sequencing

For next-generation sequencing, RNA was extracted from naso-oropharyngeal swab specimens from the second outbreak and cat K8 by using the RNAdvance Viral Kit (Beckman Coulter, https://www.beckman.com). After cDNA synthesis, sequencing was performed with SARS-COV-2 amplificons generated according to the ARTIC nCoV-2019 sequencing protocol v2 with primer version V3 (https://github.com) and subsequent library preparation with the Nextera XT Kit on the MiSeq (Illumina, https://www.ilumina.com) with 2 × 200 bp PE reads. Human samples from the second outbreak and a sample from cat K8 was sequenced by using high-throughput sequencing procedures and the Ion Torrent S5TM XL Instrument (ThermoFisher) (*8*) and subsequent enrichment by using RNA baits for SARS-CoV 2 (*9*). Sequence datasets were analyzed by using reference mapping with the Genome Sequencer Software Suite (version 2.6; Roche, https://roche.com), default software settings for quality filtering and mapping, and 2019_nCoV_Muc_IMB1 (accession no. LR824570) as a reference. Resulting SARS-CoV-2 contigs were mapped against the reference genome by using the software tool Geneious Prime (2019.2.3; https://assets.geneious.com) and gaps were filled with N to obtain full-length sequences. To identify potential single nucleotide polymorphisms in the read data and for acquisition of variant frequencies for each variant, the variant analysis tool integrated in Geneious Prime (2019.2.3) was applied (default settings, minimum variant frequency 0.02). Genome sequences assembled from cat K8 and affected residents are available in the European Nucleotide Archive Study (accession no. PRJEB41549).

References

1. Schlottau K, Rissmann M, Graaf A, Schön J, Sehl J, Wylezich C, et al. SARS-CoV-2 in fruit bats, ferrets, pigs, and chickens: an experimental transmission study. Lancet Microbe. 2020;1:e218–25. PubMed https://doi.org/10.1016/S2666-5247(20)30089-6

2. Wernike K, Aebischer A, Michelitsch A, Hoffmann D, Freuling C, Balkema-Buschmann A, et al. Multi-species ELISA for the detection of antibodies against SARS-CoV-2 in animals. Transbound Emerg Dis. 2020;Nov15:10.1111/tbed.13926. 26 PubMed

3. Jendrny P, Schulz C, Twele F, Meller S, von Köckritz-Blickwede M, Osterhaus ADME, et al. Scent dog identification of samples from COVID-19 patients: a pilot study. BMC Infect Dis. 2020;20:536. PubMed https://doi.org/10.1186/s12879-020-05281-3

4. Hoffmann B. Real-time RT-PCR specific for SARS-CoV-2 (IP4), 2020 [cited 2021 May 5]. https://www.dvg.net/fileadmin/Bilder/DVG/PDF/20-03-31-LA_RT-qPCR_SARS-CoV-2_IP4__AgPath_.pdf

5. Hoffmann B, Depner K, Schirrmeier H, Beer M. A universal heterologous internal control system for duplex real-time RT-PCR assays used in a detection system for pestiviruses. J Virol Methods. 2006;136:200–9. PubMed https://doi.org/10.1016/j.jviromet.2006.05.020

6. World Health Organization. Institut Pasteur protocol: real-time RT-PCR assays for the detection of SARS-CoV-2, March 2020 [cited 2021 May 5]. https://wwwwhoint/docs/default-source/coronaviruse/real-time-rt-pcr-assays-for-the-detection-of-sars-cov-2-institut-pasteur-parispdf?sfvrsn=3662fcb6_2

7. Corman VM, Landt O, Kaiser M, Molenkamp R, Meijer A, Chu DK, et al. Detection of 2019 novel coronavirus (2019-nCoV) by real-time RT-PCR. Euro Surveill. 2020;25:2000045. PubMed https://doi.org/10.2807/1560-7917.ES.2020.25.3.2000045

8. Wylezich C, Papa A, Beer M, Höper D. A versatile sample processing workflow for metagenomic pathogen detection. Sci Rep. 2018;8:13108. PubMed https://doi.org/10.1038/s41598-018-31496-1

9. Wylezich C, Calvelage S, Schlottau K, Ziegler U, Pohlmann A, Höper D, et al. Next-generation diagnostics: virus capture facilitates a sensitive viral diagnosis for epizootic and zoonotic pathogens including SARS-CoV-2. Microbiome. 2021;9:51. PubMed https://doi.org/10.1186/s40168-020-00973-z

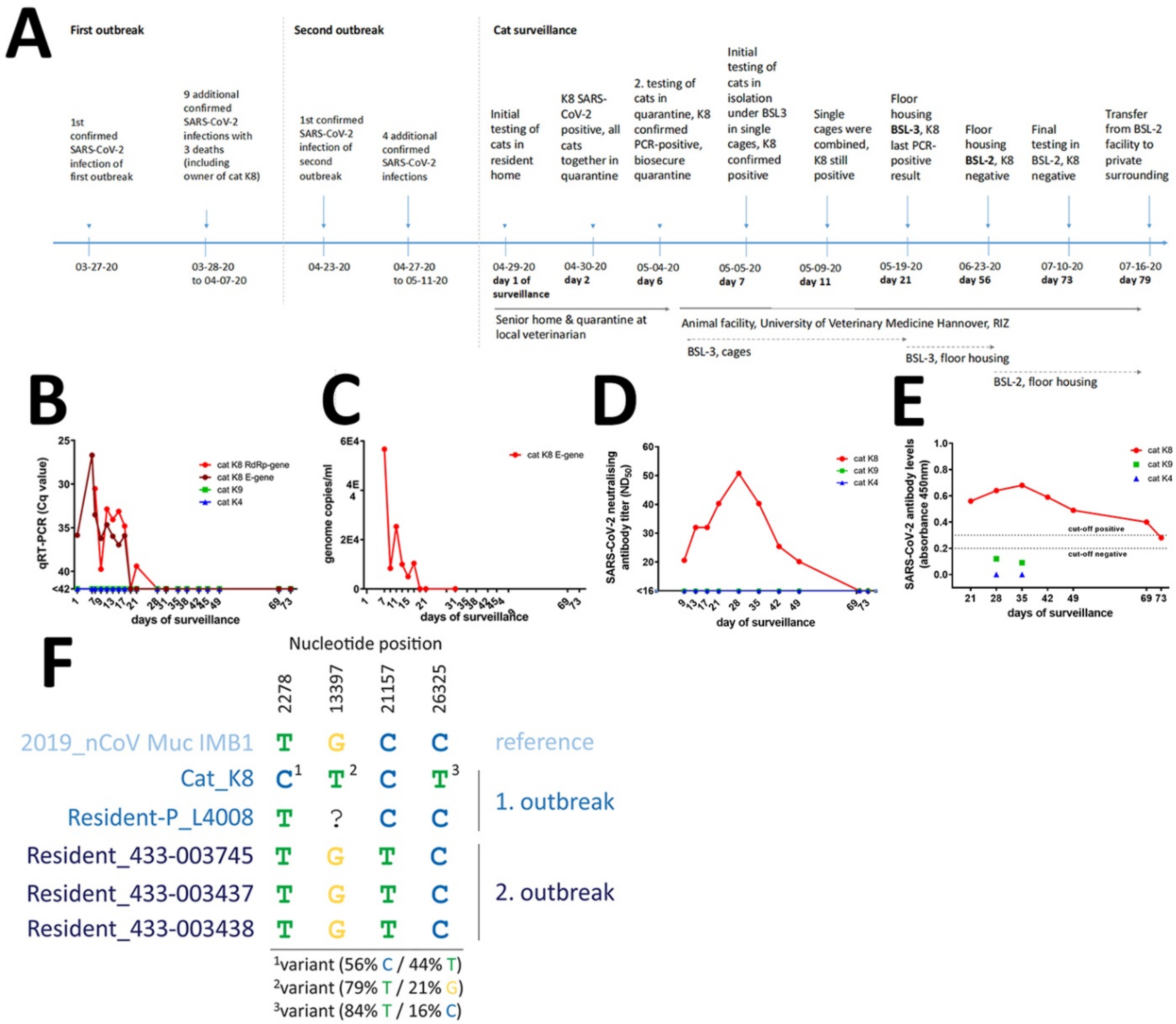

Appendix Figure 1. Human and feline monitoring after a SARS-CoV-2 cluster outbreak in a retirement home. A) Timeline of clinical events in the human SARS-CoV-2 infections, as well as virologic, serologic, and clinical surveillance of the 3 therapy cats, including their transfer for implementation of quarantine measures. BSL, biosafety laboratory; RIZ, Research Center for Emerging Infections and Zoonoses; SARS-CoV-2, severe acute respiratory syndrome coronavirus 2. B) Cq values of oropharyngeal swab specimens collected from the 3 cats during surveillance as determined by using qRT-PCR targeting either the RdRp gene (IP4 assay) or the E gene (E_Sarbeco assay). Cq, quantification cycle; E, envelope; qRT-PCR, real-time quantitative reverse transcription PCR; RdRp, RNA-dependent RNA polymerase. C) Viral RNA copy numbers per milliliter of RNA eluate from cat K8 were calculated on the basis of quantified E gene–specific RNA. E, envelope. D) SARS-CoV-2 neutralizing antibody dynamics in serum samples from 3 cats by using a neutralization test. ND_{50}, 50% neutralizing dose. E) Progression of SARS-CoV-2 antibodies specific for the receptor binding domain in indirect multispecies ELISA**.** F) Genome sequences generated from oropharyngeal swab samples (day 7) from cat K8 and affected residents. Single nucleotide polymorphism sites relative to the SARS-CoV-2 genome of isolate 2019_nCoV_Muc_IMB1

(LR824570). Nucleotide positions 2278, 13397, and 21157 are located in the open reading frame 1ab gene, and 26325 is located in the envelope gene. The question mark indicates that there is no coverage of the genome sequence at this position, genome sequences from K8 and affected residents are available in the European Nucleotide Archive Study (accession PRJEB41549).

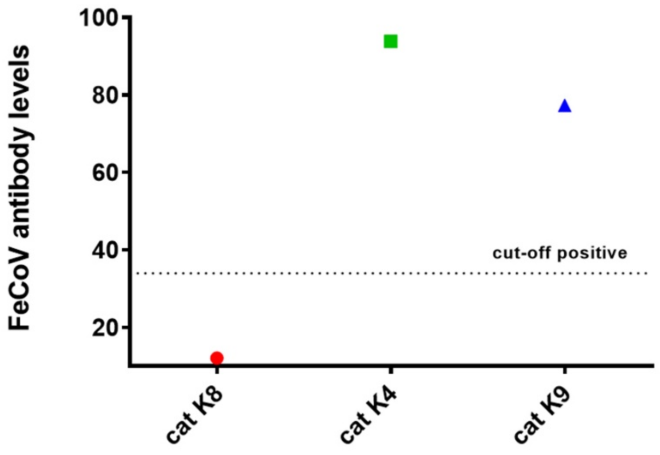

Appendix Figure 2. Prolonged SARS-CoV-2 RNA shedding from therapy cat after cluster outbreak in retirement home. Co-infection status of cats with FeCoV. FeCoV-specific IgG titers were analyzed by using a qualitative ELISA at day 28 of surveillance for the 3 cats. FeCoV, feline coronavirus SARS-CoV-2, severe acute respiratory syndrome coronavirus 2.
